# Draft Genome Sequences of *Enterobacter cloacae* Strains CAPREx E7 and CAPREx E2-2

**DOI:** 10.1128/genomeA.00488-17

**Published:** 2017-06-08

**Authors:** Rita E. Monson, Joseph Honger, Alison Rawlinson, George P. C. Salmond

**Affiliations:** Department of Biochemistry, University of Cambridge, Cambridge, United Kingdom

## Abstract

*Enterobacter cloacae* strains CAPREx E7 and CAPREx E2-2 were isolated from Ghanaian yams at a London market. The draft genome sequences indicate that the two strains are similar, with genomes of 5,042,838 and 5,039,930 bp and 56.19% and 55.05% G+C content, respectively. Both strains encoded three different β-lactamases, including one of the AmpC family.

## GENOME ANNOUNCEMENT

*Enterobacter cloacae* is a Gram-negative rod-shaped member of the family *Enterobacteriaceae*. *E. cloacae* strains are often associated with human infections, and many strains of *E. cloacae* carry β-lactamases or carbapenemases that confer antibiotic resistance (1). *E. cloacae* strains have also been isolated from plants, soil, and animals (2–5). Here, we report the genome sequences of two new strains of *E. cloacae*, CAPREx E7 and CAPREx E2-2, isolated from Ghanaian yams purchased from a London produce market. These strains were isolated from slices of yams that were grown on crystal violet pectate agar (6). Both strains were also able to grow at 30°C and 37°C on LB agar.

The draft genomes of both strains were generated by sequencing genomic DNA on Illumina MiSeq and HiSeq platforms using 2 × 250-bp paired-end reads. These reads were trimmed using Trimmomatic (7), assembled using SPAdes (8), and annotated using Prokka (9). The quality of the reads and assembly was also assessed using BWA-mem (10). The final assembly of CAPREx E7 contained 5,042,810 bp, with 56.19% G+C content, 4,712 open reading frames (ORFs), 78 tRNAs, and a mean coverage of 89.9×. The final assembly of CAPREx E2-2 contained 5,039,930 bp, with 55.05% G+C content, 4720 ORFs, 78 tRNAs, and a mean coverage of 137.07×.

*E. cloacae* strains have been isolated from many sources, and a recent source of interest has been their resistance to antibiotics and presence in persistent bacterial infections. Both CAPREx E2-2 and CAPREx E7 contained a single copy of *ampC*, a gene encoding a clinically important β-lactamase in the cephalosporinase family (1, 11). Two class B metallo-β-lactamases (MBLs) were also identified within each genome. The first, E7_03710 (CAPREx E7) or E2_03468 (CAPREx E2-2), showed high levels of similarity to a class B MBL in *Brenneria goodwinii*, a bacterium associated with oak trees (12), and *Erwinia toletana*, a bacterium associated with olive tree knots (13). The second class B MBL (E7_04150 in CAPREx E7 or E2_00247 in CAPREx E2-2) has previously been identified in many other *E. cloacae* strains but not in other plant-associated organisms.

We were also able to identify putative machinery for several secretion systems, including types I, II, IV, and VI. However, we were unable to identify any type III secretion machinery, although previous work in clinical *E. cloacae* samples found that only 27% contained type III secretion machinery (14). Secondary metabolite production can also be important in virulence. Both sequences strains contain genetic clusters predicted to encode the production machinery for the siderophore aerobactin (15, 16) and an arylpolyene similar to that produced by *Escherichia coli* (17).

The draft genomes of these two strains contain antibiotic resistance genes characteristic of other *E. cloacae* strains. However, some of their secondary metabolite clusters are most similar to those in plant-associated bacterial strains. Our hope is that these genomes will provide further help in elucidating the plant-associated life cycle of some *Enterbacter cloacae* strains.

### Accession number(s).

The draft genomes of these two strains have been deposited in GenBank under accession no. MWME00000000 (CAPREx E2-2) and MWMD00000000 (CAPREx E7).

## References

[1] Mezzatesta ML, Gona F, Stefani S 2012 *Enterobacter cloacae* complex: clinical impact and emerging antibiotic resistance. Future Microbiol 7:887–902. doi:10.2217/fmb.12.61. 22827309

[2] Yan Y, Zhao CW, Zhang YZ, Zhang ZH, Pan GL, Liu WW, Ma QY, Hou R, Tan XM 2012 Draft genome sequence of *Enterobacter cloacae* subsp. *cloacae* strain 08XA1, a fecal bacterium of giant pandas. J Bacteriol 194:6928–6929. doi:10.1128/JB.01790-12. 23209197 PMC3510591

[3] Osei Sekyere J 2016 Current state of resistance to antibiotics of last-resort in South Africa: a review from a public health perspective. Front Public Health 4:209. 27747206 10.3389/fpubh.2016.00209 PMC5042966

[4] Liu WY, Wong CF, Chung KM-k, Jiang JW, Leung FC-C 2013 Comparative genome analysis of *Enterobacter cloacae*. PLoS One 8:e74487. doi:10.1371/journal.pone.0074487. 24069314 PMC3771936

[5] Humann JL, Wildung M, Cheng CH, Lee T, Stewart JE, Drew JC, Triplett EW, Main D, Schroeder BK 2011 Complete genome of the onion pathogen *Enterobacter cloacae* EcWSU1. Stand Genomic Sci 5:279–286. doi:10.4056/sigs.2174950. 22675579 PMC3368419

[6] Perombelon MCM, Van Der Wolf JM 2002 Methods for the detection and quantification of *Erwinia carotovora* subsp. *atroseptica* (*Pectobacterium carotovorum* subsp. *atrosepticum*) on potatoes: a laboratory manual. Scottish Crop Research Institute, Dundee, Scotland http://www.scri.ac.uk/files/files/Staff/ErwiniaManual.pdf.

[7] Bolger AM, Lohse M, Usadel B 2014 Trimmomatic: a flexible trimmer for Illumina sequence data. Bioinformatics 30:2114–2120. doi:10.1093/bioinformatics/btu170. 24695404 PMC4103590

[8] Bankevich A, Nurk S, Antipov D, Gurevich AA, Dvorkin M, Kulikov AS, Lesin VM, Nikolenko SI, Pham S, Prjibelski AD, Pyshkin AV, Sirotkin AV, Vyahhi N, Tesler G, Alekseyev MA, Pevzner PA 2012 SPAdes: a new genome assembly algorithm and its applications to single-cell sequencing. J Comput Biol 19:455–477. doi:10.1089/cmb.2012.0021. 22506599 PMC3342519

[9] Seemann T 2014 Prokka: rapid prokaryotic genome annotation. Bioinformatics 30:2068–2069. doi:10.1093/bioinformatics/btu153. 24642063

[10] Li H 2013 Aligning sequence reads, clone sequences and assembly contigs with BWA-MEM. arXiv arXiv:1303.3997.

[11] Jacoby GA 2009 AmpC β-lactamases. Clin Microbiol Rev 22:161–182. doi:10.1128/CMR.00036-08. 19136439 PMC2620637

[12] Denman S, Brady C, Kirk S, Cleenwerck I, Venter S, Coutinho T, De Vos P 2012 *Brenneria goodwinii* sp. nov., associated with acute oak decline in the UK. Int J Syst Evol Microbiol 62:2451–2456. doi:10.1099/ijs.0.037879-0. 22140177

[13] Rojas AM, de los Rios JEG, Fischer-Le Saux M, Jimenez P, Reche P, Bonneau S, Sutra L, Mathieu-Daudé F, McClelland M 2004 *Erwinia toletana* sp. nov., associated with *Pseudomonas savastanoi*-induced tree knots. Int J Syst Evol Microbiol 54:2217–2222. doi:10.1099/ijs.0.02924-0. 15545461

[14] Krzymińska S, Mokracka J, Koczura R, Kaznowski A 2009 Cytotoxic activity of *Enterobacter cloacae* human isolates. FEMS Immunol Med Microbiol 56:248–252. doi:10.1111/j.1574-695X.2009.00572.x. 19527293

[15] Keller R, Pedroso MZ, Ritchmann R, Silva RM 1998 Occurrence of virulence-associated properties in *Enterobacter cloacae*. Infect Immun 66:645–649. 9453621 10.1128/iai.66.2.645-649.1998 PMC113501

[16] Van Tiel-Menkveld GJ, Mentjox-Vervuurt JM, Oudega B, de Graaf FK 1982 Siderophore production by *Enterobacter cloacae* and a common receptor protein for the uptake of aerobactin and cloacin DF13. J Bacteriol 150:490–497. 6461633 10.1128/jb.150.2.490-497.1982 PMC216393

[17] Cimermancic P, Medema MH, Claesen J, Kurita K, Wieland Brown LC, Mavrommatis K, Pati A, Godfrey PA, Koehrsen M, Clardy J, Birren BW, Takano E, Sali A, Linington RG, Fischbach MA 2014 Insights into secondary metabolism from a global analysis of prokaryotic biosynthetic gene clusters. Cell 158:412–421. 25036635 10.1016/j.cell.2014.06.034 PMC4123684

